# Non-confirming replication of “Performance of InSilicoVA for assigning causes of death to verbal autopsies: multisite validation study using clinical diagnostic gold standards,” by Flaxman et al.

**DOI:** 10.1186/s12916-020-01518-9

**Published:** 2020-03-26

**Authors:** Zehang Richard Li, Tyler H. McCormick, Samuel J. Clark

**Affiliations:** 1 Department of Biostatistics, Yale School of Public Health, New Haven, CT USA; 2 Department of Statistics, University of Washington, Seattle, WA USA; 3 Department of Sociology, University of Washington, Seattle, WA USA; 4 Department of Sociology, The Ohio State University, Columbus, OH USA

**Keywords:** InSilicoVA, Verbal autopsy, Cause of death, Reproducible research, Replication code

## Abstract

**Background:**

A verbal autopsy (VA) is an interview conducted with the caregivers of someone who has recently died to describe the circumstances of the death. In recent years, several algorithmic methods have been developed to classify cause of death using VA data. The performance of one method—InSilicoVA—was evaluated in a study by Flaxman et al., published in *BMC Medicine* in 2018. The results of that study are different from those previously published by our group.

**Methods:**

Based on the description of methods in the Flaxman et al. study, we attempt to replicate the analysis to understand why the published results differ from those of our previous work.

**Results:**

We failed to reproduce the results published in Flaxman et al. Most of the discrepancies we find likely result from undocumented differences in data pre-processing, and/or values assigned to key parameters governing the behavior of the algorithm.

**Conclusion:**

This finding highlights the importance of making replication code available along with published results. All code necessary to replicate the work described here is freely available on GitHub.

## Background

Where comprehensive systems to capture and record births and deaths do not exist, public health officials sometimes use survey-based reports to assess the distribution of deaths by cause. These are called verbal autopsies (VAs). VAs are administered to family members or caretakers of a recently deceased person, and consist of questions about the decedent’s medical history, demographics, and the circumstances surrounding their death. An additional step is required to assign a cause to each death in a VA survey.

In recent years, several algorithmic and statistical approaches for coding cause of death from VA data have been proposed. InSilicoVA is a Bayesian approach that jointly models the likely cause of death for each individual and the overall population distribution of deaths by cause. In their paper, Flaxman et al. [1] perform a comparison between InSilicoVA (developed by us) and Tariff 2.0 (developed by the authors of [1]). Both of these methods infer a likely cause of death using symptoms reported in VA surveys. The Flaxman et al. [1] paper compares these two methods using data from the Population Health Metrics Resource Consortium (PHMRC) [2]. The PHMRC dataset is unique and useful because it contains VA deaths with a medically certified reference cause, although as Garenne [3] and Byass [4] point out, it is not perfect: the deaths are coded by a single clinician, and some recorded codes are implausible.

The reported performance of InSilicoVA in Flaxman et al. [1] is notably different to previously published evaluations conducted by our group [5]. Contradictions like this risk undermining the credibility of the entire algorithmic coding enterprise, and for public health officials seeking to understand which algorithmic method may be most useful in their context, it is essential to understand what causes differences like this. In this correspondence article, we discuss how choices about data pre-processing, and differences in the information available to each algorithm, contribute to these discrepancies. We also attempt—and fail—to replicate the results presented in Flaxman et al. [1] (Table 1). Flaxman et al. [1] do not provide replication code with their article, so it is not possible to know exactly what they did.

**Table 1:** Comparison of the replication study with the published results by Flaxman et al. [1]

	InSilicoVA				Tariff 2.0	
	Our replication		Flaxman et al. [1]		Serina et al. [7]	
	Median	95% UI	Median	95% UI	Median	95% UI
CCCSMFA: no HCE	23.8	(−0.6, 48.1)	2.1	(0.5, 3.9)	23.1	(21.6, 24.3)
CCCSMFA: HCE	31.4	(6.5, 52.5)	13.9	(12.6, 15.5)	37.6	(36.5, 38.9)
CCC: no HCE	32.8	(27.2, 37.8)	28.5	(28.3, 28.7)	37.8	(37.6, 37.9)
CCC: HCE	37.9	(32.1, 43.2)	34.1	(33.9, 34.5)	50.5	(50.2, 50.7)

*CCCSMFA* chance-corrected CSMF accuracy, *CCC* chance-corrected concordance

## Flaxman et al. [1] implementation of InSilicoVA

First, we explore several decisions made by Flaxman et al. [1] when implementing InSilicoVA, which could lead to substantial performance discrepancies. Flaxman et al. [1] implemented three configurations of InSilicoVA. In the first two configurations, they map the PHMRC data into the format required for InterVA [6], another algorithm for coding cause of death using VA data. This choice greatly reduces the amount of information contained in the dataset that is available to InSilicoVA. The InterVA input format contains 245 symptoms, while only 123, 69, and 62 symptoms can be mapped from the PHMRC dataset for adults, children, and neonates, respectively. Mapping to the InterVA data structure means that InSilicoVA and Tariff 2.0 used different symptoms to assign cause of death, and the comparison in performance reflects both differences in the algorithms and differences in the data being used. There is also ambiguity about exactly how the extract.prob function was implemented in the second and third configuration. Flaxman et al. [1] claim they used the extract.prob function to obtain the empirical conditional probability matrix for all exercises—i.e., the InterVA *probbase* of Pr(*s*|*c*). The extract.prob function in the InSilicoVA package that we provide for running InSilicoVA offers three ways to calculate the conditional probability of observing each symptom given each cause: one calculates the raw conditional probability matrix, and the other two bin the elements in this matrix into *k* levels (*k*=15 by default, to be consistent with InterVA) and assign new values to cells within each level. This binning step allows the InSilicoVA algorithm to adaptively update this conditional probability matrix, to avoid being overly influenced by values close to 0 or 1. Flaxman et al. [1] do not describe what choice they made in this step.

A second potential reason for the difference in performance is the coding of missing values. Flaxman et al. [1] do not describe whether they code “missing” symptoms specifically, as instructed in the documentation of InSilicoVA package. Missing values are extremely common in VA surveys, and PHMRC is no exception— 17.8*%* missing. In addition, with more than half of the symptoms not mapped from the PHMRC data, it is not clear whether these omitted symptoms were correctly coded as “missing” instead of “absent.” A code of “missing” means that no definitive value was recorded, i.e., either the question was not asked by the interviewer, or the respondent did not know the answer to the question. “Absent,” in contrast, means that the question was asked and the response to the question was negative. In general, miscoding “missing” to “absent” changes the information contained in the data and can lead to severe underperformance of InSilicoVA.

Third, InSilicoVA was given far less information than Tariff 2.0. As described in Serina et al. [7], 677 words in the PHMRC dataset stemmed from the free-response questions. We did not include text from open-ended narrative in either McCormick et al. [5] or the replication study described below, because it requires important input from physician reviewers to remove improbable associations between stemmed words and causes. This process is described in general in Serina et al. [7]. The specific spurious associations that were removed from Tariff 2.0 were never made public, however, making it impossible for us to replicate the information available to Tariff 2.0. Further, in addition to the text information, Tariff 2.0 removes biological and epidemiologically implausible causes from the data for each observation. We implemented a similar feature for the default InterVA-type input in the InSilicoVA package, but we did not implement this when training data are used, as is the case here. The exact criterion for excluding causes was not fully explained in the literature describing Tariff 2.0, meaning—again—that Tariff 2.0 and InSilicoVA made predictions based on two fundamentally different datasets.

Finally, Tariff 2.0 redistributes the deaths assigned to indeterminate causes across all the other causes using weights from the Global Burden of Disease. InSilicoVA, in contrast, does not have a specific category for indeterminate causes and, instead, deals with indeterminate causes by spreading uncertainty across multiple possible causes. The Tariff 2.0 approach will likely improve accuracy measures if the distribution of causes in the sample of interest is similar to the Global Burden of Disease estimates. The approach used by Tariff 2.0 could be thought of as using the Global Burden of Disease cause distributions as a strong prior, such that whenever there is insufficient information in the present data to identify a cause (and, thus, it is coded as indeterminate), the Global Burden of Disease provides external information.

## Our replication study

We repeated the validation analysis conducted in Flaxman et al. [1] using publicly available software and the PHMRC reference death dataset, and we obtained very different results.

### Software

We used InSilicoVA package (version 1.1.5) and the openVA package (version 1.0.3), both the latest versions at the time of preparing this manuscript, for processing and converting the original raw dataset into binary symptoms.

### Data

We converted the original adult PHMRC gold standard data into 177 binary symptoms, as described in Section 2.2 of McCormick et al. [8]. The data transformation was based on the data cleaning procedure described in Murray et al. [2], and as discussed in McCormick et al. [8]. The conversion based on the supplemental materials from the original paper yields similar, but not exactly the same results. However, we believe that this is the closest replication of the binary symptoms possible given the publicly available information. We used the same cutoff values for continuous variables as in Murray et al. [2]. Among the three configurations used in Flaxman et al. [9], our data processing steps are perhaps most similar to the third configuration described as “with empirical probbase matching Tariff 2.0,” with a few differences discussed below.

A more important potential difference between our replication study and that of Flaxman et al. [1] is the use of “health care experience” (HCE) variables. Since the authors presented the InSilicoVA experiment performance with and without HCE variables without defining them, we turn to the definition provided in Serina et al. [7], where questions related to (1) history of chronic illness and (2) interaction with health services, and all text from open-ended narratives are classified as HCE variables. In our implementation of InSilicoVA, we only used the first group of symptoms: 14 symptoms related to history of chronic conditions, e.g., “Did decedent have asthma?” We performed 2 sets of analysis with and without these 14 symptoms. It is unclear if this is the same interpretation of “HCE” and “no HCE” as in Flaxman et al. [1].

Finally, as discussed above, Tariff 2.0 assigns indeterminate causes to some deaths, as described in Serina et al. [7], and then redistributes those to all other causes using weights from the Global Burden of Disease. It is not clear if Flaxman et al. [1] performed the same procedure, or if they did, how the re-weighting using this external information affects the performance metrics.

### Method and parameters

We implemented the InSilicoVA method by sampling from the posterior distribution of all parameters using Markov Chain Monte Carlo for 10,000 iterations. We discarded the first 5000 iterations as burn-in, and saved every tenth iteration in the second half. For the proposal distribution in the Metropolis-Hastings step, we set jump.scale =0.5. This option leads to an acceptance rate of around 0.3 for this analysis.

### Replication code

All software and code [10] used in this study are free and publicly available, see the “Availability of data and materials” section below.

### Results

Table 1 presents results using InSilicoVA from our replication study, and that of Flaxman et al.[1], and the comparison to the results using Tariff 2.0, which was originally published in Serina et al. [7]. Compared to our replication, the performance metrics for InSilicoVA presented by Flaxman et al. [1] are far lower and have implausibly narrow uncertainty intervals. This strongly suggests that Flaxman et al. [1] did not correctly implement InSilicoVA in their tests. Because Flaxman et al. [1] do not fully describe how they prepared the data, and because they do not provide replication codes, no one can know for sure what they did.

## Additional comments

There are several additional issues in Flaxman et al. [1] that require clarification.

First, the chance-corrected concordance (CCC) metric was described as having a range of [−1,1], with − 1 indicating no ability to detect a cause. However, the definition of the CCC [9] reveals that it takes a value of − 1/(*N*−1) when no deaths are correctly classified, where *N* is the number of causes. Obviously, this value is equal to − 1 only when there are exactly two causes. In all other circumstances, the lower bound of CCC is not equal to − 1. This misrepresentation of the range of the CCC creates confusion for readers.

Second, the interpretation of the chance-corrected CSMF accuracy (CCCSMFA) in Flaxman et al. [1] is misleading. The correction term (1−*e*^−1^) is calculated based on the limiting expectation of the CSMF accuracy metric as the number of causes of death (rather than the number of observations) increases to infinity, under a particular assumption about the true CSMF vector, namely that it follows a Dirichlet distribution with parameter 1. For any given true underlying CSMF, one can imagine accounting for random cause allocations when comparing to that particular truth. However, it is not clear why it is necessary to adjust the CSMF accuracy using an unrealistic hypothetical limiting value. Flaxman et al. [1] describe the characteristics of the CCCSMFA to include “values near 0.0 indicate that the diagnostic procedure being applied is essentially equivalent to random guessing.” The mismatch between the actual underlying CSMF and the hypothetical reference CSMF means that this statement is not necessarily true. One trivial counterexample is when the true CSMFs include equal fractions for all causes. In this situation, random guessing of individual cause of death should yield perfect CSMF estimates and, in return, CCCSMF accuracies close to 1.0. No other CSMF estimator can outperform random guessing, as it coincides with the true data-generating process at the population level, and a value of 0.0 in CCCSMF only means that the CSMF accuracy is at an arbitrary value of 0.632. This clearly illustrates the potential misinterpretation of the metric when the reference CSMF is mistakenly thought of as representing “random guessing.” In fact, since the CCCSMFA is simply a fixed linear transformation of the much more widely used CSMF accuracy, it adds no information beyond CSMF accuracy. We underscore the importance of ensuring this new metric does not lead to misinterpretations.

## Discussion and conclusion

In this correspondence article, we reveal and, insofar as possible, explain discrepancies in the performance of the InSilicoVA method presented in Flaxman et al. [1], compared to similar results published previously by our group [5, 8]. Using publicly available data, we attempted and failed to replicate the evaluation study in Flaxman et al. [1]. We speculate on several possible explanations, but because the codes to conduct the Flaxman et al. [1] evaluation are not available, we cannot be certain. Perhaps, the most important conclusion from this work is that transparent replication codes must be published with the substantive results, and the data used for a study must be made available—otherwise, it is not possible to truly interrogate and thereby trust the results. Vague—and, very often, woefully incomplete—summaries of the analysis are completely inadequate. While the review process at *BMC Medicine* is quite transparent, with reviewer names and their reviews being made public, truly transparent science requires replication code and access to data. The choices made by Flaxman et al. [1] in their evaluation, combined with our inability to replicate their results, are a noteworthy example of the importance of making full code available.

We conclude with a discussion of additional points raised by Flaxman et al. [1] regarding the InSilicoVA method. Flaxman et al. [1] claim that InSilicoVA has many customizable parameters. The method is designed to work when specifying only a number of iterations, and a tuning parameter controlling the sampling acceptance rate. Additional parameters are available to ensure that a user understands all of the choices being made when implementing InSilicoVA, but it is not necessary to change their values to successfully use the method. We note that having few user-facing parameters is not the same as making few consequential choices about the data analysis process, and we believe that revealing the choices is far better than hiding them. For example, under the simple user interface of the SmartVA-Analyze application, there are several fixed or hidden parameters in Tariff 2.0 [7]. These include the number of bootstrapped samples, the resampling algorithm, the threshold values used to remove impossible causes at the individual level, and the re-weighting of indeterminate deaths. All these parameters have important impacts on the results, and users may benefit from setting them to values different from the supplied defaults.

On page 9 of Flaxman et al. [1], the authors claim that using expert-derived conditional probabilities as inputs does not distinguish between presenting and reporting a symptom. We agree that the VA interview is a major source of uncertainty, and believe that more work should be done to understand reporting. Unfortunately, “gold standard” reference deaths, like the PHMRC data used by Tariff 2.0 (SmartVA-Analyze software), have consequential limitations as well; for example, they do not represent the majority of deaths for which VA is useful, i.e., deaths that happen at home and not in a hospital. Consequently, by definition, algorithms trained using such deaths cannot be assumed to work well for community deaths.

Flaxman et al. [1] point out that the InSilicoVA framework gives a warning if the method does not converge. In this context, convergence refers to the process used to take samples from the posterior distribution that is required to obtain estimates. This arises because InSilicoVA uses a Bayesian approach. The Bayesian approach is advantageous because it permits sharing of uncertainty between individual causes of death and population distributions. The downside is that the method requires additional computation. We include this warning so that a user is aware they might need to run additional iterations to obtain appropriate estimates, especially for the causes with small CSMFs, not in general to indicate that the method has failed.

It is worth noting that the InSilicoVA algorithm was thoroughly evaluated and vetted by referees who are experts in statistical methodology prior to its publication in a reputable statistics journal, the *Journal of the American Statistical Association* [5, 8]. As far as we can determine, none of the various versions of the Tariff algorithm have been rigorously examined in this way by reviewers with the specific expertise to evaluate their structure and performance. Typical public health and medical practitioners do not have the in-depth knowledge or experience necessary to thoroughly interrogate mathematical, statistical, machine learning, or other computational methods.

The final recommendation of this work is that complex mathematical, statistical, or computational methods applied to health-related questions should first be evaluated and certified by appropriately skilled experts in the relevant fields before they are used and published in public health or other medical studies. We conclude that users of VA algorithms should be very careful in interpreting comparisons that purport to show superior performance of one method over another.

## Data Availability

The data used in this study are freely available for download from the Institute for Health Metrics and Evaluation [11]. The R codes used to replicate this analysis are publicly available on GitHub [10].

## Abbreviations

CCC: Chance-corrected concordance
CCCSMF: Chance-corrected cause-specific mortality fraction
CSMF: Cause-specific mortality fraction
HCE: Healthcare experience
PHMRC: Population Health Metrics Research Consortium
UI: Uncertainty interval
VA: Verbal autopsy
